# On Mr. Want's Discovery of the Mode of Preparing the Eau Medicinale

**Published:** 1814-09

**Authors:** Thomas Sutton

**Affiliations:** Croom's Hill, Greenwich


*For the Medical and Physical Journal.*

### *On Mr. Want's Discovery of the Mode of preparing the Eau Medicinale;* by Dr. Sutton.

I OBSERVE, in your last Number (for July), a paper written by Mr. Want on the cure of the Gout, by a medicine which he conceives to be the true eau medicinale. It certainly is a creditable effort in any gentleman to endeavour to discover of what a secret medicine, of acknowledged usefulness and powers, consists; as the public ought to have more real confidence in a medicine whose properties are known, and which the regular faculty can recommend, than in one which has not acquired such a sanction. But if it should happen that this secret medicine, whatever it may be, should be proved to possess no greater efficacy than many known remedies, then the real value of the discovery is much diminished, and it becomes a mere matter of curiosity. I consider the discovery of the composition of the French nostrum, at the present time, exactly in this point of view, because I am confident that we possess a numerous class of medicines which are as equally capable of subduing a paroxysm of gout as the eau medicinale. I much doubt, however, whether Mr. Want has arrived at the discovery of this last medicine, though I am far from concluding that his paper is devoid of interest on that account; for I am fully convinced that he has announced a medicine which is capable of effecting all he has stated. The value of Mr. Want's communication, I judge, consists in announcing the cure of forty patients in the gout by a known medicine, and by exciting an action* which has been before stated to be capable of subduing the paroxysms of this disorder. It also announces the use of a drug which has not been of late employed for the purpose, if it ever was at any time; but the latter, in my opinion, is by no means so important to establish as the result of the prac-

* This case shews decidedly that a regular fit of the gout may be rapidly cured by exciting the intestines to powerful action, and that this desirable object may be accomplished by the aid of known remedies. *Vide* Tracts on Delirium Tremens, Gout, &c. by Thomas Sutton, M.D. page 206, bottom of the page.

To subdue a paroxysm of the gout, it must be observed that the operation (of cathartics) should be powerful; and, although we may not be able to show the exact reason of this, it must be kept in mind that attention to this point is the material circumstance to be relied upon for complete success in subduing the paroxysms of gout. *Idem*, p. 208, bottom of the page.

tice.

tice. This remedy is announced by Mr. Want with a great degree of candour; and, though it may be as eligible as the eau medicinale, I judge it not to be one so generally applicable as might be wished. It may be that the drug called hermodactyl by Trallian may happen to be a very different one to that which we have used under that name;* and, as he appears to ascribe to it a more powerful cathartic operation than we find in the modern hermodactyl, Mr. Want's medicine may therefore come nearer in effect to the drug used by Trallian. But this neither proves that the colchicum autumnale is the hermodactylus of Trallian, nor that the eau medicinale is prepared from this drug. We indeed find, that he recommends scammony to be occasionally added to his medicine, in order to obtain a complete evacuation of the bowels, as may be seen by Mr. Want's quotation, which one might presume it would not need if the hermodactyl of Trallian was the real colchicum autumnale.

Several years ago I recommended a preparation of elatorium and opium to imitate the operation of the eau medicinale, and it was not only found to be equally effectual, but to operate in the same precise manner. A gentleman to whom I prescribed this medicine, in the dose of two grains to sixty drops of laudanum, and who had frequently taken the eau medicinale, told me that the sensations excited in him were precisely the same as those occasioned by the French nostrum. But the object that always seemed of the most importance to me on this subject, was not what the French medicine consisted of, so much as by what operations it produced its salutary effects. These appeared to me to be effected, in the most material and most permanent degree, by a powerful action on the bowels, although it certainly more immediately allays the violence of pain by its anodyne quality. Having come to this conclusion, if accurate, we have laid open to us a numerous class of medicines, which

* Alston considers this to be the case. (*Vide* Alston's Materia Medica, article Hermodactylus.) But as it is very evident that the colchicum autumnale could not be the hermodactyl of Turner, as his prescription directs fifteen grains at a dose, with equal parts of other purgatives, (*vide* Allen's Synopsis Medicinæ, art. Gout;) and Stœrck, who paid much attention to the operation of colchicum autumnale, states, that a quantity of less than a grain, wrapped up in crumbs of bread, and taken internally, produced alarming symptoms. A strong infusion of the linum catharticum, or purging flax, has been used by several persons in this neighbourhood, in gout, with success. This medicine produces very copious actions on the bowels.

may may effect equally beneficial purposes. This I have stated in my Tract on Gout; and I never, in curing an ordinary paroxysm of this disease in a healthy subject, think so much of the sort of purgative it is necessary to give, as of the doses which will induce the quantum of operation I wish to arrive at.

By keeping this in view, Epsom salts, aloëtic purgatives, calomel joined with other purgatives, jalap, gamboge, and other cathartics, given in such doses as to produce a powerful action on the intestines, may each accomplish all that is necessary, which, when this has been effected, may be followed by an anodyne at night; and these proceedings have caused as much benefit as I have ever heard the eau medicinale was capable of doing. The inquiry, therefore, having assumed this form, we are able to select from a large class of purgative medicines what may appear to be the most eligible for the various conditions of the disease and of constitution, and be able to attend to those idiosyncrasies that we occasionally meet with in persons under this disorder.

Thus far I can aver in the cure of gout, or an attempt to ameliorate the symptoms, that there is no state, short of that in which death appears to be quickly approaching, in which something may not be beneficially done by a prudent use of purgatives, and by having the whole class of them thrown open for selection. But if, on the contrary, the proper virtues of purgatives for the cure of gout can only be supposed to reside in such as the French medicine, or in such drugs as the colchicum autumnale, or as the elaterium, or as hellebore, then we should find numerous instances in which we would rather allow nature to take her course, than endeavour to controul the gout by medicines which might promote a very powerful effect on feeble constitutions, on irritable stomachs, and on other conditions in respect to age, disease, habit, &c.

By the proper use of purgative medicines in numerous instances of gout, I have seen the paroxysms of this disease overcome in the short space of a few hours, and the entire restoration of the limb affected to follow in a few days: in others, where all this benefit could not be expected to ensue, I have observed the pain to be quickly subdued, and the patient to return to a better state of limbs than before the attack of the disease. In only one instance have I found the powers of purgatives to subdue a paroxysm of gout, and a perfect restoration not to follow, where it might have been expected. This case was also as little benefitted by the eau medicinale. The patient had, however, the satisfaction to find an increase of healthful feelings, by pursuing the plan laid down in my Tract on Gout, though the paroxysms, although though ameliorated, continued to return. It is too early yet to judge how far the plan laid down for this patient may be wanting in ultimate success. We ought not, however, to be discouraged by a few cases not suffering all the controul that appears to be capable of being effected in a great majority; nor ought those to detract much from the important views in which the cure of gout has of late years been placed; nor should the benefit capable of being attained by many be disregarded, because it cannot be extended to all. In the case alluded to, there are circumstances which evidently separate it from the ordinary and concurring symptoms of the gout, though, on a superficial view of the disease, it would be difficult to consider it to belong to any other species of disorder.

*Croom's Hill, Greenwich,* THOMAS SUTTON, M.D.

*July* 4, 1814.

---

Mr. Want *in answer to* Dr. Sutton.

The preceding observations are made with a view to depreciate my discovery of the mode of preparing the Eau Medicinale, because, as Dr. Sutton says, he is confident we possess a numerous class of remedies which are as capable of subduing a paroxysm of gout as the eau medicinale. Were this assertion supported by the experience of the profession, my discovery would, I admit, be of no value. If salts, magnesia, and rhubarb, will cure gout, as Dr. S. would have us believe, there can be no necessity for having recourse to a potent and deleterious drug, as mine is. But I can affirm, from repeated experiment, that no one of this numerous class of remedies deserves the character bestowed upon them. The doctor may be very correct when he says that Epsom salts and aloes have done every thing *he has heard* the eau medicinale is capable of doing; for, to adopt his own language, if it should *happen to be* that he is proved to be totally unacquainted with the curative properties, or the sensible effects of that remedy, the efficacy of these medicines may not be much overrated.

The infallibility of this class of medicines is maintained throughout the whole paper, but the sentiment is so diffused that it is difficult to fix upon a sentence sufficiently decisive to afford matter fit for quotation. The author, indeed, in one place distinctly asserts that there is no state short of that in which death is actually approaching, in which something may not beneficially be done by the prudent use of purgatives; and such has been the success of his practice, that in only one instance has he found the powers of purging to subdue a paroxysm of gout without a perfect restoration following; and he never thinks so much of the *kind* of purgative, as of the *quantum* of operation necessary to be induced. After having established that *all purgatives* will cure gout, the doctor proceeds to show that my medicine operates by exciting an action (on the bowels) which had before been stated to be capable of curing the disease. Now, the legitimate inference from these premises would be, that, although I did not deserve to be immortalized for the discovery, yet my medicine, being a purgative, was at least capable of curing the gout. But Dr. S. draws a very different conclusion, for although, he observes, the medicine may be equally eligible with the eau medicinale, yet he judges it not to be so extensively applicable as might be wished. Let it, however, be understood, that I quarrel with this conclusion only, as being inconsistent with what the author had previously maintained as indisputable facts, for it requires no peculiar sagacity to discover that this or any other known remedy is not so universally applicable as might be wished. I have made no promises of infallibility; I simply state that this medicine does that which the eau medicinale is capable of performing; and I recommend it to those only who have experienced the good effects of that remedy.

That it is not so extensively applicable as could be wished, may be collected from my own paper, wherein I have expressly adverted to its bad properties. I have there stated, that in some instances its employment may be attended with consequences fatal to the patient, particularly when administered without regard to the peculiar circumstances of the case. That this expression implies the existence of a case in which it is inapplicable, is a proposition that surely cannot be denied.

The assertion that all purgatives will cure gout, is so obviously unsupported by common experience, that our readers will scarcely require its refutation; and it only remains for me to show, that the curative powers of the colchicum autumnale are quite unconnected with its purgative operation. Upon this I may observe, that in many cases it removes the paroxysm of gout without *any sensible operation of any kind*. This fact is *very notorious*. If Dr. S. requires the proof of it, I may instance (among many others) the case of the illustrious President of the Royal Society, long known to have been a martyr to the disease. Sir Joseph Banks assures me, that in him the eau medicinale never produces any action on the bowels, while it never fails to relieve him; and he farther states, that the accidental occurrence of purging will generally bring on a fit of the gout, and, if present, materially aggravate the complaint. But this seems to arise from a peculiarity of constitution not constantly met with; though we are not without other examples of the same kind. It was probably the same idiosyncracy which induced Sydenham so strongly to deprecate the employment of purgatives under any circumstances in gout. Lucian, in his poetical and very accurate account of this disease, intimates the tendency of purging to produce or aggravate the fit, when he makes the goddess Podagra say, she would fall with greater fury upon those who purged themselves with the hiera picra. These instances, however, have not made me insensible to the value of that class of remedies, for, in the month of August 1811, I wrote an essay for the Med. and Phys. Journal expressly for the purpose of recommending them, *not as infallible*, but as being most extensively useful. The elaterium, which Dr. S. akes to himself the credit credit of introducing into practice, though his Tract did not appear till the year 1813, was first recommended by *me* in 1811, accompanied with cases of success from its employment, and it may be remarked that one case is there related where it failed,* as purgatives frequently do; and the patient has since my discovery been invariably relieved, and the paroxysm removed, by the colchicum.

It cannot be denied, as *I have shewn in my former essay*, that the drastic purgatives, (the foremost among which is elaterium,) are, if properly administered, very useful in the treatment of this complaint; but they have failed, and will fail in a multitude of cases, perfectly within the influence of the meadow saffron.

Dr. Sutton says, the hermodactyl described by Trallian *may happen to be* a very different medicine from that which we use under that name. If it *only may happen to be*, it *may not be*, different. If the doctor had any doubts respecting it, it was incumbent upon him to state the reasons which induced him to differ. He assumes indeed that Alexander ascribed to the hermodactyl a more powerfully purgative operation than we find in the modern hermodactyl. I am at a loss to conceive what foundation there can be for this opinion, when we are expressly told by this author, as Dr. S. unaccountably admits, that even in cases where a large dose of his medicine seems to have been prescribed, if a fuller evacuation from the bowels was desirable, scammony was to be added.

Ει δε θελεις ᾽επι πλεον υπαγειν την γαστερα προσμιγνυε σκαμμωνιας κε.δ. και αλυπως καθαιρει και ανωδυνους ποιει τους πασχοντας.

Alex Tralian, cap. xi.—περι ανωδυνων αντιδοτων και φαρμακων καθαρτικων.

Here appears no evidence of extraordinary purgative powers possessed by the hermodactyl, and, admitting that it sometimes occasioned the watery evacuation from the bowels described in my last, yet we are led to conclude there were cases where it failed to produce this effect, and where the addition of stronger purgatives was rendered necessary. It cannot escape notice that from a part of this quotation (καθαιρει αλυπως) it appears that even the addition of so much scammony (16 grains) was supposed to render it but a *mild purge*. In my experiments with the eau medicinale, colchicum, and the modern imported hermodactyl, I find they are also severally very variable, and equally uncertain in their operation, which seems to be governed by constitutional peculiarity, so much so that in scarcely two persons have they precisely the same effect This fact has been universally remarked with respect to the Eau Medicinale; and it is so striking in the other two as to add, in my opinion, very considerably to the evidence of their identity. In the case of Mr. Wallis, of Judd-street, now under my care, a full dose of the Tincture of Colchicum produced a sickness with vomiting, which continued to harass him for twenty-four hours, and yet this extreme dose produced no purgative operation whatever. I have witnessed the same effect so frequently, that I have no hesitation in maintain-

* Of John Tomkin Great St. Andrew-street, Seven Dials.

ing, that in a multitude of cases, if given in a dose just sufficient to cure the patient, and no more, it will be found to exert no purgative quality whatever, and very little sensible operation of any kind. This has probably been observed by those writers on the materia medica who affirm that the modern hermodactyl has scarcely any purgative power, and who, from an erroneous idea of the stronger purgative qualities of the ancient medicine, are from this circumstance induced to believe them essentially different. But, if we extend our researches, even among the ancients we shall find the same virtue and the same power ascribed to their hermodactyl. In the compositions of the Arabian writer, Mesue,* it is generally, if not invariably, mixed up with drastic purgatives, which generally constitute the greater bulk of the compound. Dr. S. refers to Alston, in support of his opinion; but this writer has furnished us with a striking quotation from Mesue, which refutes himself. The Arabian physician tells us, in the most unequivocal terms, that the hermodactyl, (the ancient,) *if unmixed with other medicines, is scarcely a purgative*:—"per se enim tarde et imbecille vacuat." Paulus Ægineta, who is said to have practised about fifty years after Trallian, tells us the hermodactyl moves the bowels, but does not lead us to expect strong purgative effects from it.†

Thus we find the same degree of power ascribed to both the modern and ancient drug by the writers of their respective times; and, if we attentively investigate the subject, we shall discover also the same contradictions and the same implications of the uncertainty of their operation, which I have discovered to be equally characteristic of the meadow saffron and the eau medicinale. There seems then no reason to believe them to be different medicines, from the supposed discrepancy between the modern and ancient writers.

Impressed with the belief, that the modern hermodactyl possesses less purgative powers than that described by the ancients, Dr. S. conjectures that the colchicum autumnale, on account of its stronger operation, may more nearly resemble the drug prescribed by Trallian. That it does not only resemble Trallian's drug, but is the same medicine, is a fact of which I do not entertain the smallest doubt; but I do not discover this resemblance in the stronger purgative operation it is supposed to be endued with. I have before denied that there is any foundation for believing this strong purgative power to be an *invariable* property of the ancient hermodactyl, from which, if correct, it will follow that this supposition of our author is entirely gratuitous. In the case of Mr. Wallis, before adverted to, it had no such operation, though given in an *extreme dose*. Upon what authority, I would ask, does the doctor suppose this plant to be possessed of that quality? Certainly not from his own personal observation, or we should never have had the account of Storck's expe-

* *De Medicamentis simplicibus et compostis.*

† Hermodactyli radix et per se & ipsius decoctum habet vim purgandi. privatim arthriticis tunc, cum humores defluunt exhibetur, verum stomacho nimis quam adversatur.

riment riment upon himself,* brought as an evidence on the occasion, a story more ridiculous and monstrous than any to be found in Baron Munchausen, and which can only be equalled by the countless falsehoods contained in his several publications.

The writers on the materia medica are generally silent as to this purgative effect of colchicum.† The English dispensatories who quote from Storck never hint at it. I have not time at present to peruse Storck's book, (which I have never yet thought of sufficient importance to procure, on account of his notorious inaccuracies;) but it is reasonable to suppose, that if he had seen it exert a purgative operation, he would have named it, and they would have copied him. I am not acquainted with any writer who has himself seen this effect; but the little time allowed to an answer of this kind in a public Journal, will not permit me to enquire very minutely into the circumstance. Dioscorides, as well as some modern authors, are of one accord as to its poisonous qualities. The former of these says, the colchicum, if *eaten*, like the fungi, kills by strangulation, but never mentions purging:

Βρωθεισα δε κλεινει καλα πνιγμον ομοιως μυκησιν.

Geoffry, who seems to be unacquainted with the properties of the plant from personal observation, says, "it is related that those who *eat it* experience itchings throughout the whole body, tearing pains in the bowels, with heat and weight in the stomach. As the symptoms increase, blood is voided by stool, and the colchicum comes away in pieces."‡ I have seen cases in which it produced a most alarming

---

* Storck was a German physician of extensive reputation, the disciple and contemporay of the great De Haen.—De Haen, in his hospital, before a numerous body of students and practitioners, tried in vain to produce the same curative results from cicuta, one of Storck's panaceas, which Storck professed to have witnessed, and challenged him publicly to demonstrate to the medical students of the university the truth of his allegations respecting it—*it was never done.* De Haen investigated the fact alleged by his former pupil respecting the cure of thirty-six cases of cancer, and found thirty of them had died the victims of the disease, and six remained uncured. See his Epistola de Cicuta.

In a cotemporary publication, written by Storck or his friends, entitled "Alethophilorum quorundam Viennensum—Elucidatio necessaria Epistolæ de Cicuta," De Haen's conduct was ascribed to envy; but whatever be the motive, the fact is undoubted: for the falsity of all Storck's publications on materia medica is quite proverbial.

† I am indebted to Sir Joseph Banks for a neat epigram on the colchicum, the two last lines of which are appropriate to our subject:

Illinor arthesi utiliter—solorque podagram

At ne me *mandas* ni cupis mori.

The last idea is literally transcribed from Dioscorides.

‡ I find this is copied from Dioscorides under the article Ephemerum, which he calls Colchicum. The term has probably been given to it from the supposition of its producing death *in one day.*

 sense sense of suffocation, from the globulus hystericus and flatulent distension of the abdomen, without purging. Though more commonly, in a full dose, it has this effect; which if it does take place, immediately removes the sense of suffocation. This is one of the circumstances in which I have observed, as referred to in my last, such coincidence in effect between the hellebore (which rarely purges) and this medicine.

One author only, Ludovicus, relates, that a single root of colchicum almost killed a patient *by purging*. Garidel, in his Histoire des Plantes des environs d'Aix, records, that a servant whom he attended was killed by taking the flowers for an intermitting fever, in which it is said to be useful. The symptoms were probably those mentioned above, which he terms "anxietés et des tranchés pendant trois jours;" no purging is mentioned. The Turks are said to intoxicate themselves with the flowers macerated in wine.*

Prosper Alpinus says the colchicum is perfectly inert, and that the Ægytian women fatten themselves with the *roasted* roots, often eating twenty in the course of the day, without any evacuation from the stomach or bowels.† But let it be observed, these roots were *roasted*, the process of which may materially alter their properties. Thus the onion, an acrid, stimulating, pungent root, when roasted loses all these qualities, and the same is true of many other plants that could be named.

Cartheuser, misled by this quotation, says, that the plant growing near him *also* has neither diuretic or cathartic power, nor does it possess any noxious property.—" *Id compertum habeo* nostri pa-

---

* *Geoffry, Materia Medica*, vol. vi.

† " Mulieres Ægyptiæ, *scribit*, multa factitant in balneis, ut pingues evadant.—Comedunt etiam adhuc multa, gallinas quippe arte pingues factas, nucemque Indicam in jure dissolutam, ac in massam redactam. Sed ex omnibus pro secreto habent singulo die, dum eunt dormitum, ad decem vulgares bulbos, pro hermodactylis a nostris Phamacopœis receptos, quos aliqui potius Colchicum esse autumant, contostos mandere, eosque pluribus diebus, quindecim scilicet & viginti ad usque frequentant. Ex quorum usu, quod nostris mirum videbitur, nihil vel per alvum, vel vomitum, evacuant, minusque aliâ molestiâ mulieres vexantur, &c."—*De Medicinâ Ægyptiorum, Lib. III. Cap. XVI. pag.* 109.

Again:—" Mulieres pauperculæ, sumptum pro aliis ferre nequeuntes, vulgares hermodactylos, quibus communiter nostri pharmacopœi utuntur, modicè contostos, æque atque nos castaneas edimus multos unicâ vice, ad impinguescendum, devorant, ex quibus neque alvus aliquo pacto turbatur, neque aliud quippiam mali accedit. Hinc nostri pharmacopolæ scire possunt, quantum, illis pro vero hermodactylo utentes, hactenus erraverint. Egoque hos non parum admiratus sum, quando Ægyptiæ mulieres earum radicum (quas sine dubio si modo Dioscoridi credendum sit, Colchici esse quisque herbarum materiæ peritus fatebitur) per multos dies, ad decem & plures etiam, euntes dormitum sumpsisse, instarque castanearum comedisse, sine ullâ noxâ, ibi sæpius compererim, &c."—*Lib. IV. Cap. I. pag.* 118.

it riter colchici communis, in Marchia et Silesia superiori crescentes radices nec *catharsin nec largiorem diuresin* concitari nec hominibus etiam lethiferas et saltem noxias existere."*

A difference of soil and climate *might* produce a variation in the strength of this drug, but it will be easy to account for the contradictory statements of authors respecting it by the singular diversity of its operation in different constitutions. The opinions of Alpinus may be clearly traced to the fact of his colchicum having been roasted, and others *(cæci cæcos sequentes)* have copied him without taking the pains to inquire whether he was right or wrong. Its history is one of the most striking instances of the careless manner in which medical investigations are for the most part carried on. Here is a plant possessing peculiar powers, and is described as far back as the time of Dioscorides, and by most, if not all, succeeding writers on materia medica; and yet these properties have invariably escaped observation by those whose business, as authors, it ought to have been to have ascertained its effects.

The identity of the tincture of colchicum and eau medicinale, is a question which can only be determined by attentive examination, and comparison of their respective operations on the human body. I will pledge my professional reputation for the truth of what I have alledged on this subject. My practice in gout has been very great, and, where these remedies appeared likely to be useful, I have administered them with the most careful observation of their effect, and have never once entertained a doubt of their being the same medicines. I am assisted in forming my judgment by the testimony of those who have taken both medicines. I have had intercourse with many of the most distinguished literary characters of this kingdom, who are conversant both with the appearance and properties of the French remedy; and they are *unanimous* in expressing their convictions that the two compositions are identically the same. When considering the question of identity, it may be useful to advert to the fact that one Wedelius, a continental physician, sold an empirical preparation of colchicum, which, like the French nostrum, was extolled as a panacea. This, indeed, is so very common with advertized medicines, that I should not think the circumstance worth notice, if the catalogue of its virtues did not bear some resemblance to that which we find in Husson's original advertisement. It is also deserving of remark, that the account of this nostrum is contained in a System of Materia Medica (by Geoffry) well known in France, where Huson lived.

In the hurry of drawing up this answer, I may have possibly omitted some particulars important to have been mentioned. Should this hereafter appear to be the case, I shall willingly resume the subject: at present I shall merely add, that my convictions respecting these medicines are unalterably fixed.

*No. 9, North Crescent,* JOHN WANT,

*Bedford-square.* *Surgeon to the Northern Dispensary.*

* *Cartheuser Materia Medica*, vol. ii.

